# Prognostic factors for survival of patients with nasopharyngeal carcinoma following conventional fractionation radiotherapy

**DOI:** 10.3892/etm.2013.1100

**Published:** 2013-05-08

**Authors:** FEI KONG, BAI-ZHEN CAI, XIAN-ZHAO CHEN, JIAN ZHANG, YI-MING WANG

**Affiliations:** Department of Radiotherapy, The People’s Hospital of Hainan Province, Haikou, Hainan 570311, P.R. China

**Keywords:** nasopharyngeal carcinoma, conventional fractionation radiotherapy, prescription dose, tumor stage

## Abstract

This study aimed to investigate the risk factors influencing the prognosis of patients receiving conventional fractionation radiotherapy. A retrospective analysis of clinical data from 100 patients with nasopharyngeal carcinoma receiving radiotherapy was conducted. The Chi-square test was used to screen the relevant factors and Cox multiple-factor analysis was used to investigate the risk factors influencing the survival of patients. One-factor analysis results revealed that tumor stage, tumor diameter, prescription dose completion and radiotherapy regularity are related to the prognosis of nasopharyngeal carcinoma and multiple-factor analysis results revealed that tumor stage, radiotherapy dose and radiotherapy regularity are independent risk factors influencing prognosis. The prognosis of patients with nasopharyngeal carcinoma receiving radiotherapy is related to tumor progression and an adequate dose of regular radiotherapy improves the prognosis of patients.

## Introduction

Nasopharyngeal carcinoma is the most common malignant tumor in the head and neck region and 80% of nasopharyngeal carcinoma cases worldwide occur in China. The incidence of nasopharyngeal carcinoma significantly increases in individuals aged >30 years and 80% of cases occur in individuals aged 30–60 years (1–3). Nasopharyngeal carcinoma is sensitive to radiotherapy and the short-term treatment efficacy is good; however, the recurrent rate and the distant metastasis rate remain high. Therefore, the prognosis varies (4–7). Clinical studies have confirmed that among patients with nasopharyngeal carcinoma receiving radiotherapy, tumor progression is rapid and clinical prognosis is poor, even if patients begin radiotherapy immediately (8,9). We conducted a retrospective analysis of clinical data from patients with nasopharyngeal carcinoma receiving radiotherapy in our hospital to investigate the relevant factors influencing the prognosis and survival of patients, as well as the independent risk factors, to provide a reference for the evaluation of nasopharyngeal carcinoma radiotherapy and prognosis.

## Materials and methods

### Clinical data

A total of 100 patients with nasopharyngeal carcinoma who received radiotherapy in the Radiotherapy Department in our hospital from January 2006 to December 2006 were selected. There were 62 male and 38 female cases, aged 18–78 years. The mean age was 51±18.6 years. Diagnoses of all patients were confirmed by pathological examination. According to the standard of nasopharyngeal carcinoma diagnosis and treatment prepared by the Otolaryngology Branch of Chinese Medical Association (in 1992, Fuzhou, China), there were 91 cases in stage I, 24 cases in stage II, 36 cases in stage III and 21 cases in stage IV. Among them, there was 1 case of carcinoma sarcomatodes and 99 cases of poorly-differentiated squamous cell carcinoma. Additionally, there were 68 cases with completed prescription dose radiotherapy, 27 cases with severe radiotherapy complications and 65 cases that received regular radiotherapy. This study was approved by the ethics committee of People’s Hospital of Hainan Province (Haikou, China). The informed consent was obtained from all patients.

### Radiotherapy

Prior to therapy, computed tomography (CT), magnetic resonance imaging (MRI) and electroconvulsive therapy (ECT) examinations were performed for all patients in order to evaluate the foci. With the exception of patients with lesion metastases, all other patients received targeted conventional fractionation radiotherapy using a Varian 23EX-518 medical linear accelerator (6 MV X-ray and 9 MeV β-ray). The primary clinical target area encompassed the gross tumor volume (GTV) plus a 5–10-mm extension, and the lymphatic drainage area encompassed the GTV plus a 3-mm extension. The outlined target area data were processed by the treatment planning system (TPS) to prepare the radiotherapy plan and prescription dose. The radiotherapy dose for the primary foci and cervical lymph node metastasis foci was 60–70 Gy and the prophylactic neck dose was 50 Gy, fractionated 30 times for irradiation. The non-target area and sensitive organs were shaded and the received dose was no more than the tolerance dose. In addition, CT scanning was conducted every 2 weeks to evaluate treatment efficacy, and the radiotherapy range and ray dose were determined again.

### Factor analysis and assignment method

The clinical data of patients were sorted and classified. Data for analysis included gender (male compared with female), age (patients over the mean age compared with patients under the mean age), tumor stage, tumor diameter (tumors with diameter greater than the mean compared with those with diameter less than the mean), radiotherapy dose (patients who completed the prescription dose compared with patients who did not), radiotherapy regularity (patients who received regular compared with irregular radiotherapy) and radiotherapy complications (patients experiencing complications compared with those with no complications). Survival time was recorded according to the follow-up records for patients. The cases lost to follow-up were excluded.

### Statistical analysis

Data were tabled using Excel and processed using the Chinese version of SPSS 17.0 statistical software. Survival time was analyzed by the Kaplan-Meier test, the relevant factors were screened by chi-square test and independent risk factors were analyzed by the Cox proportional hazards model. α= 0.05 was considered to indicate a statistically significant difference.

## Results

### Survival time and survival rate

Kaplan-Meier survival analysis results revealed that the median survival time of patients with nasopharyngeal carcinoma receiving radiotherapy was 52 months. The 1-, 3- and 5-year survival rates were 93.0, 80.0 and 73.0%, respectively.

### Analysis of prognosis-related factors

Among the 100 patients with nasopharyngeal carcinoma receiving radiotherapy, there were 62 male and 38 female cases. Of these, 56 cases were <51 years old and 44 cases were ≥51 years old. The tumor diameter was <2.4 cm in 42 cases and ≥2.4 cm in 58 cases. Furthermore, 19 cases were stage I, 24 cases were stage II, 36 cases were stage III and 21 cases were stage IV. In addition, 68 cases completed prescription dose radiotherapy, 65 cases received regular radiotherapy and 27 cases presented severe radiotherapy complications. The results of one-factor analysis revealed that gender, age and severe radiotherapy complication of patients were not significantly associated with the prognosis of patients (all P>0.05), while tumor stage, tumor diameter, prescription dose completion and radiotherapy regularity were significantly associated with the prognosis of patients (all P<0.05). The results are presented in Table I. We identified that a tumor diameter ≥2.4 cm, an advanced stage, uncompleted prescription dose and regular radiotherapy are factors related to a poor prognosis of nasopharyngeal carcinoma radiotherapy.

### Risk factors

According to one-factor analysis, we identified that four factors affect prognosis. Therefore, a logistic model was induced. Cox multiple-factor analysis results revealed that nasopharyngeal carcinoma tumor stage, prescription dose completion and radiotherapy regularity were independent risk factors influencing the survival of patients. Advanced tumor stages increase the risk of mortality (P=0.004). In addition, completed prescription dose (P= 0.003) and regular radiotherapy (P=0.002) significantly reduce the risk of mortality. The results are presented in Table II.

## Discussion

There are numerous nasopharyngeal structures and nasopharyngeal carcinoma is the most common malignant tumor in the nasopharynx. Radiotherapy is the main treatment method for nasopharyngeal carcinoma. For the majority of nasopharyngeal carcinomas, radiotherapy improves the clinical result. Clinical studies have confirmed that following radiotherapy in patients with nasopharyngeal carcinoma, the 5-year survival rate reaches 50–80% (10–12). Although radiotherapy produces an ideal result for the majority of nasopharyngeal carcinomas, the median survival time for patients with nasopharyngeal carcinoma receiving radiotherapy in this study was 52 months and the 5-year survival rate was 73.0%, consistent with previous results (13–15). Despite the improved clinical efficacy observed in a number of cases of nasopharyngeal carcinoma, tumor recurrence and metastasis are the main causes of treatment failure.

Among certain individuals, prognoses of radiotherapy are inconsistent. Therefore, it is important to evaluate the prognosis of patients prior to radiotherapy. One clinical study (16) confirmed that the clinical stage of nasopharyngeal carcinoma is closely related to the prognosis. Furthermore, the tumorous metastasis stage is an independent risk factor influencing prognosis and is also the main influence on short-term efficacy for nasopharyngeal carcinoma. According to the data in the present study, the prognosis of patients with nasopharyngeal carcinoma receiving radiotherapy is not associated with age, gender or radiotherapy complications; however, tumor stage, tumor diameter, prescription dose completion and radiotherapy regularity are associated with prognosis.

In nasopharyngeal carcinoma, the onset age range is larger and young patients are common. However, we did not identify an association between age and prognosis. The main cause of nasopharyngeal carcinoma onset is nasopharyngeal viral infection; however, previous studies did not identify a correlation between nasopharyngeal carcinoma and sex hormones. Additionally, tumor diameter and stage of nasopharyngeal carcinoma are related to prognosis, suggesting that tumorous biological behavior remains one of the main influences on the prognosis of patients with nasopharyngeal carcinoma receiving radiotherapy (10,17,18).

For nasopharyngeal carcinoma with a large volume and an advanced stage, the possibility of tumorous residues following radiotherapy is greater and these are the main causes of tumor recurrence following treatment. If a poorly differentiated nasopharyngeal carcinoma is recurrent, its highly malignant biological behavior usually causes rapid progression of the tumor, resulting in a poor prognosis. Among radiotherapy-related factors, prescription dose completion and radiotherapy regularity are related to prognosis, while radiotherapy complications are not related to prognosis. The common complications of radiotherapy for the treatment of nasopharyngeal carcinoma include adjacent neural injury, radiation dermatitis of the head and neck and oral mucosa injury. These often affect the quality of life of patients.

With the development of studies highlighting radiotherapy, the possibility of fatal injury to the spinal cord and other important organs is lower. Therefore, if patients complete the radiotherapy plan, there are fewer influences on prognosis. In the treatment of nasopharyngeal carcinoma with radiotherapy, completed planned prescription dose and regular radiotherapy are vital (19,20). The prescription dose is the load dose required to remove tumor tissues and cells in the target area to the maximum extent. Regular radiotherapy achieves the expected efficacy of the prescription dose.

The results in this study demonstrate that the malignant biological behavior, progression and radiotherapy implementation according to the plan of nasopharyngeal carcinoma are the factors influencing the prognosis of patients with nasopharyngeal carcinoma receiving radiotherapy. Therefore, for patients with nasopharyngeal carcinoma receiving radiotherapy, it is necessary to accurately evaluate tumor progression and strictly implement radiotherapy according to the plan to achieve an improved clinical efficacy.

## Figures and Tables

**Table I.** Analysis of factors related to the prognosis of patients with nasopharyngeal carcinoma radiotherapy.

Factors	n	No. of survivors			P-value
		1 year	3 years	5 years	
Gender					
Female	38	37	35	32	>0.05
Male	62	56	45	41	
Age (years)					
≥51	44	37	36	34	>0.05
<51	56	56	50	39	
Tumor diameter (cm)					
<2.4	42	41	38	35	<0.05
≥2.4	58	47	43	38	
Cancer stage					
I	19	19	16	16	<0.05
II	24	24	22	22	
III	36	35	30	30	
IV	21	15	13	5	
Prescription dose completed					
Yes	68	66	63	62	<0.05
No	32	27	18	11	
Regular radiation					
Yes	65	64	60	57	<0.05
No	35	29	21	16	
Serious radiotherapy complications					
Yes	27	24	19	14	>0.05
No	73	69	62	59	

**Table II.** Analysis of independent risk factors for survival of patients receiving radiotherapy for nasopharyngeal carcinoma.

Factors	β-value	Standard error	Wald value	OR value	95% CI	P-value
Cancer stage	0.675	0.214	10.140	1.796	0.599–3.106	0.004
Prescription dose radiotherapy	−0.724	0.269	7.926	0.692	1.412–2.969	0.003
Rule radiation	−0.892	0.304	7.108	1.549	0.826–2.742	0.002

OR, odds ratio; CI, confidence interval.
